# Use of Patient-Specific Instrumentation (PSI) for glenoid component positioning in shoulder arthroplasty. A systematic review and meta-analysis

**DOI:** 10.1371/journal.pone.0201759

**Published:** 2018-08-22

**Authors:** Guillaume Villatte, Anne-Sophie Muller, Bruno Pereira, Aurélien Mulliez, Peter Reilly, Roger Emery

**Affiliations:** 1 Service d'Orthopédie-Traumatologie, Hôpital Gabriel Montpied, Clermont Ferrand, France; 2 Université Clermont Auvergne, SIGMA Clermont CNRS, UMR 6296, Clermont-Ferrand, France; 3 DRCI, CHU de Clermont Ferrand, Clermont Ferrand, France; 4 Bioengineering Department, Imperial College, London, United Kingdom; 5 Division of Surgery, Imperial College, London, United Kingdom; University of Memphis, UNITED STATES

## Abstract

**Introduction:**

Total Shoulder Arthroplasty (TSA) anatomical, reverse or both is an increasingly popular procedure but the glenoid component is still a weak element, accounting for 30–50% of mechanical complications and contributing to the revision burden. Component mal-positioning is one of the main aetiological factors in glenoid failure and thus Patient-Specific Instrumentation (PSI) has been introduced in an effort to optimise implant placement. The aim of this systematic literature review and meta-analysis is to compare the success of PSI and Standard Instrumentation (STDI) methods in reproducing pre-operative surgical planning of glenoid component positioning.

**Material and methods:**

A search (restricted to English language) was conducted in November 2017 on MEDLINE, the Cochrane Library, EMBASE and ClinicalTrials.gov. Using the search terms “Patient-Specific Instrumentation (PSI)”, “custom guide”, “shoulder”, “glenoid” and “arthroplasty”, 42 studies were identified. The main exclusion criteria were: no CT-scan analysis results; studies done on plastic bone; and use of a reusable or generic guide. Eligible studies evaluated final deviations from the planning for version, inclination, entry point and rotation. Reviewers worked independently to extract data and assess the risk of bias on the same studies.

**Results:**

The final analysis included 12 studies, comprising 227 participants (seven studies on 103 humans and five studies on 124 cadaveric specimens). Heterogeneity was moderate or high for all parameters. Deviations from the pre-operative planning for version (p<0.01), inclination (p<0.01) and entry point (p = 0.02) were significantly lower with the PSI than with the STDI, but not for rotation (p = 0.49). Accuracy (deviation from planning) with PSI was about 1.88° to 4.96°, depending on the parameter. The number of component outliers (>10° of deviation or 4mm) were significantly higher with STDI than with PSI (68.6% vs 15.3% (p = 0.01)).

**Conclusion:**

This review supports the idea that PSI enhances glenoid component positioning, especially a decrease in the number of outliers. However, the findings are not definitive and further validation is required. It should be noted that no randomised clinical studies are available to confirm long-term outcomes.

## Introduction

The number of shoulder arthroplasties has been constantly increasing since the beginning of the century [1–3], even faster than lower extremity joint replacements lately [4]. This is especially the case with anatomic Total Shoulder Arthroplasty (aTSA) and reverse Total Shoulder Arthroplasty (rTSA) [2,5,6].

The glenoid component is generally regarded as the more problematic in both aTSA (loosening and wear) and rTSA (loosening and notching), accounting for up to 30–50% of overall complications [7]. This is due to two main parameters: inadequate glenoid bone stock and deformities [8,9]; and component mal-positioning [10,11] (excessive retroversion or inclination and glenoid vault perforation). The latter results in abnormal loading of glenoid areas [12] and may alter stress in the cement mantle [13].

Improvements have been reported thanks to the use of 3D-planning (compared to 2D-planning) [14,15], but the surgeon’s ability to reproduce the plan is limited due to multiple factors (surgeon’s accuracy, complex glenoid deformities and no reliable intra-operative landmark). Consequently, Computer-Assisted Surgery (CAS) and Patient-Specific Instrumentation (PSI) were introduced. CAS is accurate and reliable but its drawbacks (costs, additional steps and operating time) limit its use [15–17]. PSI is a newer technique in the shoulder (first commercialised in 2013) and many major prosthesis companies have by now developed their own philosophy and promoted solution. Developments in this area have resulted in a custom-made guidewire for the positioning of the glenoid component. A few weeks before the surgery, the surgeon either directly conducts the pre-operative planning on dedicated software with 3D glenoid reconstruction images from a CT scan, or modifies a proposed plan provided by engineers. Once the planning is validated by the two parties (surgeon and company), the 3D-printed model of the glenoid and the personalized guide-wire are made, then sent to the surgeon.

PSI is an example of the evolution towards personalised treatment that occurs in all fields of medicine [18,19]. Short and long-term benefits of this technique in knee surgery are well known [20–23] but its real impact in shoulder arthroplasty is not clear.

The aim of this systematic literature review and meta-analysis is to evaluate the efficacy of PSI to reproduce pre-operative surgical planning of the glenoid component positioning. The hypothesis is that PSI should provide better glenoid positioning than Standard Instrumentation (STDI).

## Materials and methods

This work was conducted and reported in accordance with PRISMA (Preferred Reporting Items for Reviews and Meta-Analysis) (S1 Table). The protocol was validated and registered to PROSPERO (CRD42018099761).

### Data sources and search strategy

The search was conducted on four databases: MEDLINE via PubMed, the Cochrane Library, EMBASE and ClinicalTrials.gov (for on-going trials). The last search was performed on the 1^st^ January 2018 (S1 Text).

Relevant reports were identified using the keywords: “Patient-Specific Instrumentation (PSI)”, “shoulder”, “glenoid”, “arthroplasty”, “Standard Instrumentation (STDI)”, and “free-hand”. A search algorithm was developed for each database, without any limit on publication period. The reference list of each article or report identified by the search and any previously published review on the topic were examined.

We included all studies reporting results about glenoid component positioning after use of PSI during TSA, whether the report was published, unpublished, or in press. Exclusion criteria were: no CT-scan analysis results for the component positioning, studies about TSA revisions, case reports, studies done on plastic bones, use of a reusable or generic guide, and previous reviews. Relevant trials were selected by two of the authors (GV and ASM), who worked independently from each other and resolved disagreements by consensus. Excluded trials were listed, detailing the reasons for exclusion.

### Data extraction and synthesis

Data extraction was done independently by two authors (GV and ASM). The following data was collected: identifying information (first author and year of publication); details of the study protocol and design, type of patient (human or cadaver); type of TSA (anatomic or reverse); type of PSI and pre-operative surgical planning software (automated or manual); type of pre-operative glenoid morphology (native version and inclination) and final glenoid component or pin position (version, inclination, entry point, rotation and 3D orientation), based on CT scan analysis. Then, they were organized into an Excel spreadsheet for analysis.

### Risk of bias

Two reviewers (GV and ASM) evaluated the quality of the selected studies independently without blinding for authorship or journal. For the randomized studies, the risk of bias was evaluated using the Cochrane Risk of Bias Tool [24]. The quality items assessed were selection bias (random sequence generation, allocation concealment), performance bias (blinding of patients and investigators), detection bias (blinding of outcome assessors), attrition bias (incomplete outcome data), reporting bias (selective reporting) and other forms of bias (significantly different group comparisons, funding sources, early termination of a trial). For the non-randomized studies, the quality was assessed using the Methodological Index for Non-Randomized Studies (MINORS) [25]. The index uses eight categories (for non-controlled studies) and twelve (for controlled studies) to evaluate the different kinds of bias. The items are scored as 0 (not reported), 1 (reported but inadequate) or 2 (reported and adequate), with the global ideal score being 16 for non-controlled studies and 24 for controlled studies.

Disagreements were resolved by consensus. Publication bias was assessed using the funnel plot technique.

### Main outcomes and measures

The primary outcome of this analysis is a comparison between the efficacy of PSI and STDI methods to reproduce pre-operative planning (based on a glenoid component’s deviation from planning with respect to version, inclination, entry point and rotation).

Secondary outcomes are: the assessment of PSI accuracy and reliability to reproduce pre-operative planning (based on a component’s deviation from planning with respect to version, inclination, entry point and rotation); and the number of outliers (defined as more than 10° of deviation from the planning for version or inclination or more than four millimetres away in any direction from the planned entry point).

### Statistical analysis

After extraction, all analysis was conducted using the Comprehensive Meta-Analysis software (version 2; Biostat, Englewood, NJ). Data included sample size, mean and standard deviation for each parameter, in addition to details about the study (did the study use PSI, was it a cadaveric or clinical study). The standardized means were calculated using a random-effects model (DerSimonian and Laird approach), which accounts for true variation in effects occurring from study to study and for random errors within a single study. The random-effects model was preferred to a fixed-effect model as certain experimental parameters had wide variation. The I^2^ index was used to measure heterogeneity with 25%, 50% and 75% indicating low, moderate and high heterogeneity, respectively. Finally, funnel plots [26] were used to assess publication bias. In the absence of bias, studies should be distributed evenly around the mean effect size because of random sampling error.

## Results

### General results

**Fig 1:**
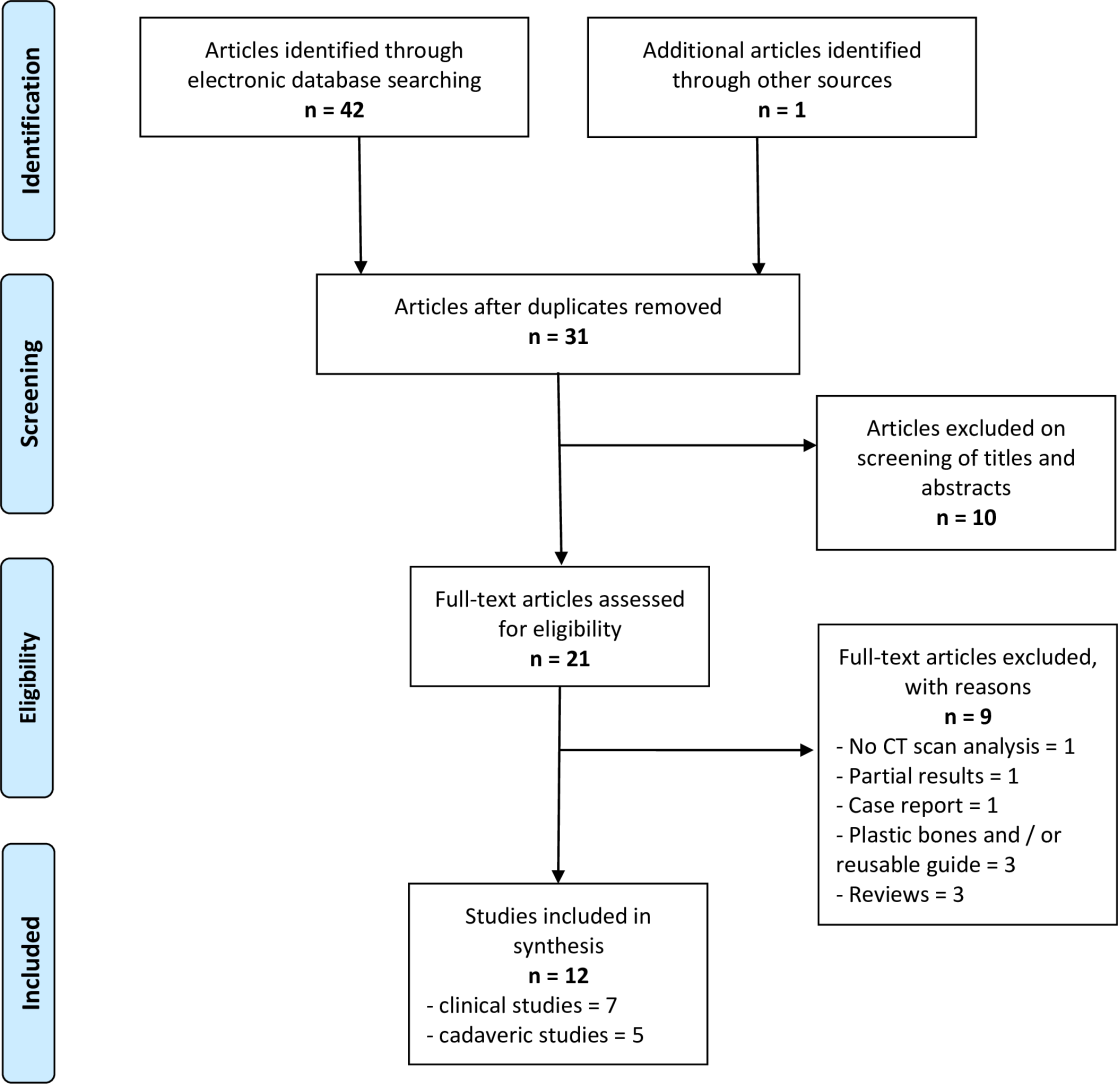
PRISMA flow-chart of study selection.

The literature search identified a total of 43 articles. Among them, 12 were identified as relevant studies according to inclusion/exclusion criterion, comprising a total of 227 cadavers or patients (Fig 1). Seven of these studies were clinical [27–33] involving a total of 103 patients. Of these seven studies, one was a prospective randomized study directly comparing PSI and STDI glenoid component positioning results [28] (involving 31 patients) and six were non-controlled studies reporting only results with PSI [27,29–33] (involving 72 patients). Of the five studies [34–38] carried out on 124 cadaveric shoulders, two were controlled studies comparing PSI and STDI [34,36] with a total of 80 subjects and three were non-controlled studies [35,37,38] with a total of 44 subjects (reporting only results with PSI).

The reported outcomes were deviation from the planning for version and inclination in all studies, from the entry point in 10 studies (five cadaveric and five clinical studies) [27–30,33–38] and from the rotation in three studies (one cadaveric and two clinical studies) [28,33,34]. Seven studies also reported the number of outliers (three cadaveric and four clinical studies) [28,29,31,32,36–38]. Reaming depth has never been reported. All CT scans were performed in the early post-operative period (hence no long-term data has been taken in to consideration).

All the procedures were carried out through a delto-pectoral approach, and no adverse events or problems linked to PSI were reported. The final goal of the procedure was to implant an anatomic glenoid component in eight studies [27–30,32,36–38] (128 patients or cadavers) and a reverse glenoid component in eight studies [29–32,34–36,38] (89 patients or cadavers).

Two kinds of processes for pre-operative surgical planning were used. The first was based on a fully automatic software performing 3D reconstruction and glenoid measurement calculations, followed by planning conducted by the surgeon. For the second process, the 3D reconstruction and measurements needed manual assistance from a technician or an engineer from the company. A planning proposal was then submitted to the surgeon, who could potentially modify it.

**Fig 2:**
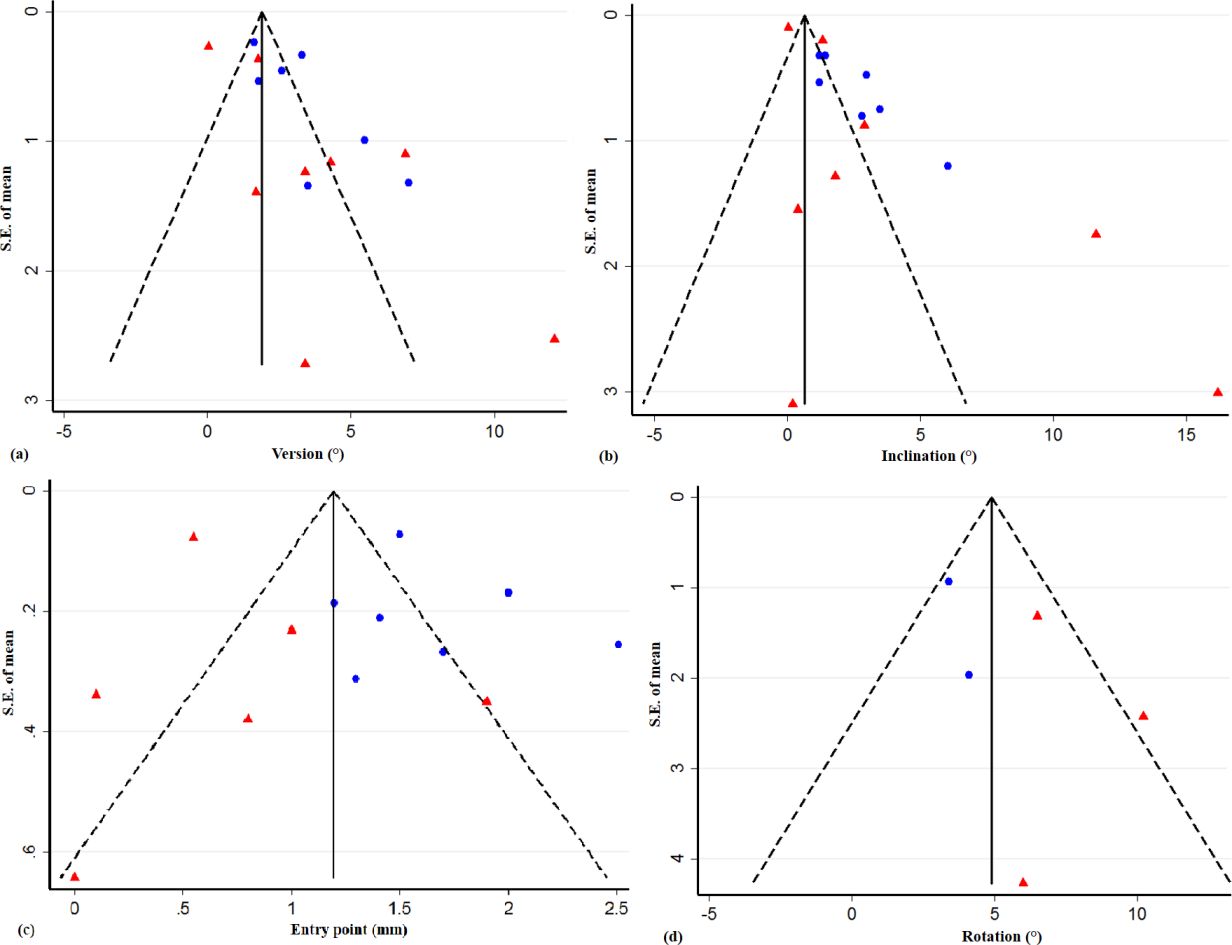
Funnel plots to assess the risk of publication bias (blue points = cadaveric studies; red triangles = clinical studies).

Table 1. shows the potential levels of bias (which were acceptable), and the funnel plot (Fig 2) assesses the risk of publication bias. The shape of the funnel plot could indicate an important publication bias, but could also be due to the heterogeneity of the included studies and poor methodological design of the smallest studies.

**Table 1:** Methodological quality of included studies, with an evaluation of bias.

**First author–Date**	**Country**	**Type of study**	**Evaluation of risk of bias**												
			**Methodological Index for Non-Randomized Studies (MINORS)**												
			It. 1	It. 2	It. 3	It. 4	It. 5	It. 6	It. 7	It. 8	It. 9	It. 10	It. 11	It. 12	Total score
Suero et al– 2012	USA	Clinical non controlled	1	2	2	2	2	2	1	0	NA	NA	NA	NA	12
Pietrzak – 2013	USA	Cadaveric non controlled	1	1	1	1	2	2	2	0	NA	NA	NA	NA	10
Subrama- nya et al—2014	Australia	Clinical non controlled	2	1	1	1	2	2	2	0	NA	NA	NA	NA	11
Levy et al– 2014	USA	Cadaveric non controlled	1	1	2	1	0	2	2	0	NA	NA	NA	NA	9
Walch et al– 2014	France	Cadaveric non controlled	2	0	2	1	2	2	2	0	NA	NA	NA	NA	11
Trockmorton et al– 2015	USA	Cadaveric controlled	2	2	2	2	2	2	2	2	2	2	2	2	24
Eraly et al– 2016	Belgium	Cadaveric controlled	2	1	2	2	2	2	2	0	2	2	1	2	20
Gauci et al– 2016	France	Clinical non controlled	2	2	2	2	2	2	2	0	NA	NA	NA	NA	14
Dallalana et al– 2016	Australia	Clinical non controlled	1	2	2	1	2	2	2	0	NA	NA	NA	NA	12
Lau et al– 2017	Australia	Clinical non controlled	1	2	2	1	2	2	2	0	NA	NA	NA	NA	12
Berouhet et al– 2018	France	Clinical non controlled	2	2	2	1	2	2	2	0	NA	NA	NA	NA	13
			**Cochrane Risk of Bias Tool (for randomized studies)**												
			It. 1		It. 2		It. 3		It. 4		It. 5		It. 6		It. 7
Hendel et al– 2012	USA	Clinical Randomi-sed	2		2		2		2		2		2		1

The items are scored 0 (not reported), 1 (reported but inadequate) or 2 (reported and adequate). The MINOR index uses eight categories (for non-controlled studies) and twelve (for controlled studies) to evaluate the different kinds of bias, the global ideal score being 16 for non-controlled studies and 24 for controlled studies. NA = Not Applicable.

### Outcomes results (Table 2)

**Fig 3:**
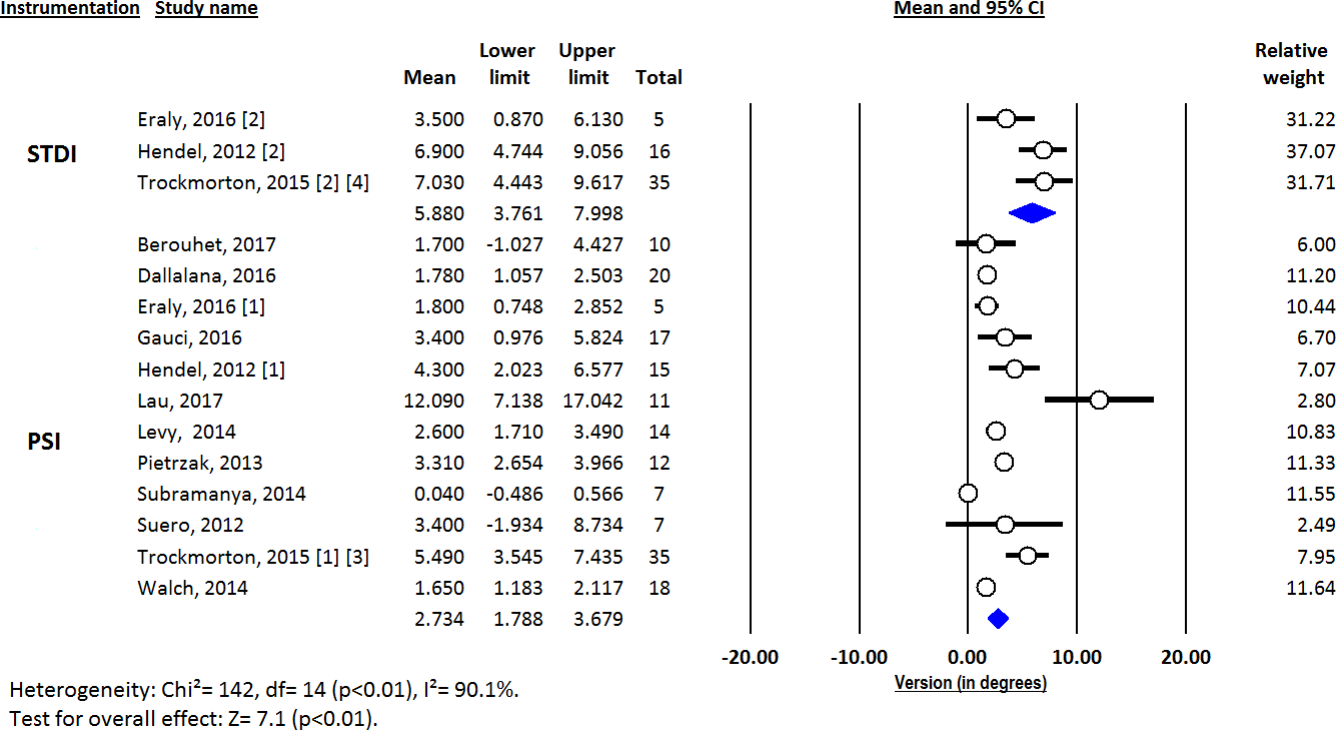
Forest plot of version deviation from the pre-operative planning, for all included studies.

**Fig 4:**
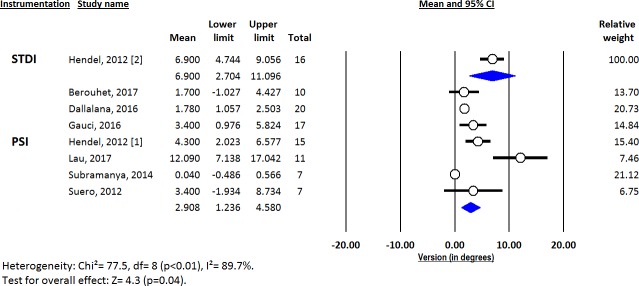
Forest plot of version deviation from the pre-operative planning, for clinical studies.

**Fig 5:**
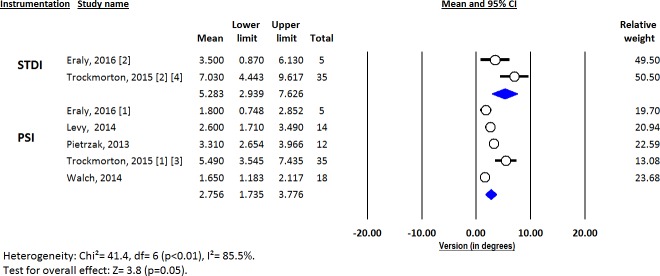
Forest plot of version deviation from the pre-operative planning, for cadaveric studies.

**Fig 6:**
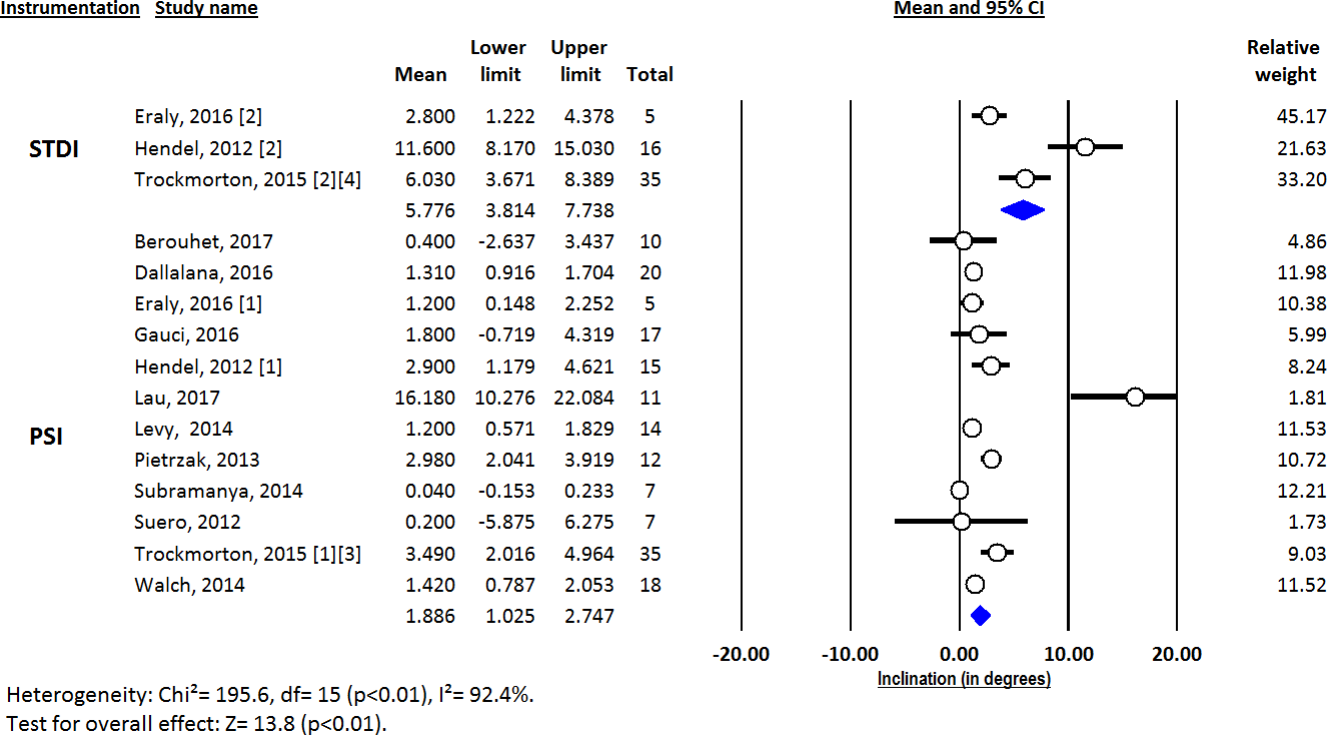
Forest plot of inclination deviation from the pre-operative planning, for all included studies.

**Fig 7:**
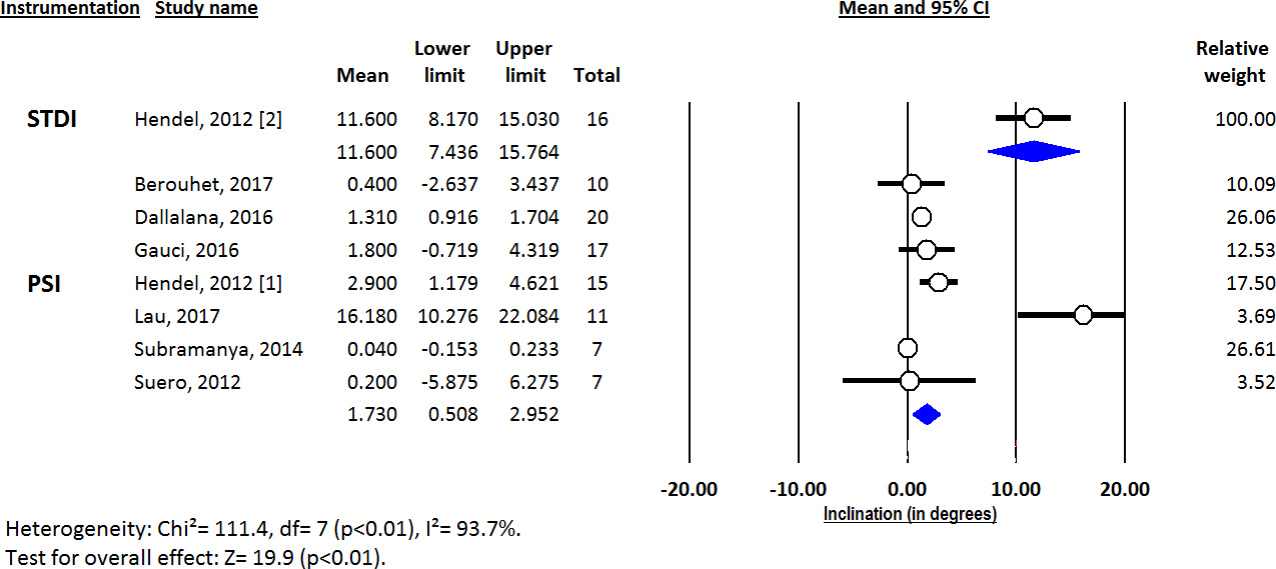
Forest plot of inclination deviation from the pre-operative planning, for clinical studies.

**Fig 8:**
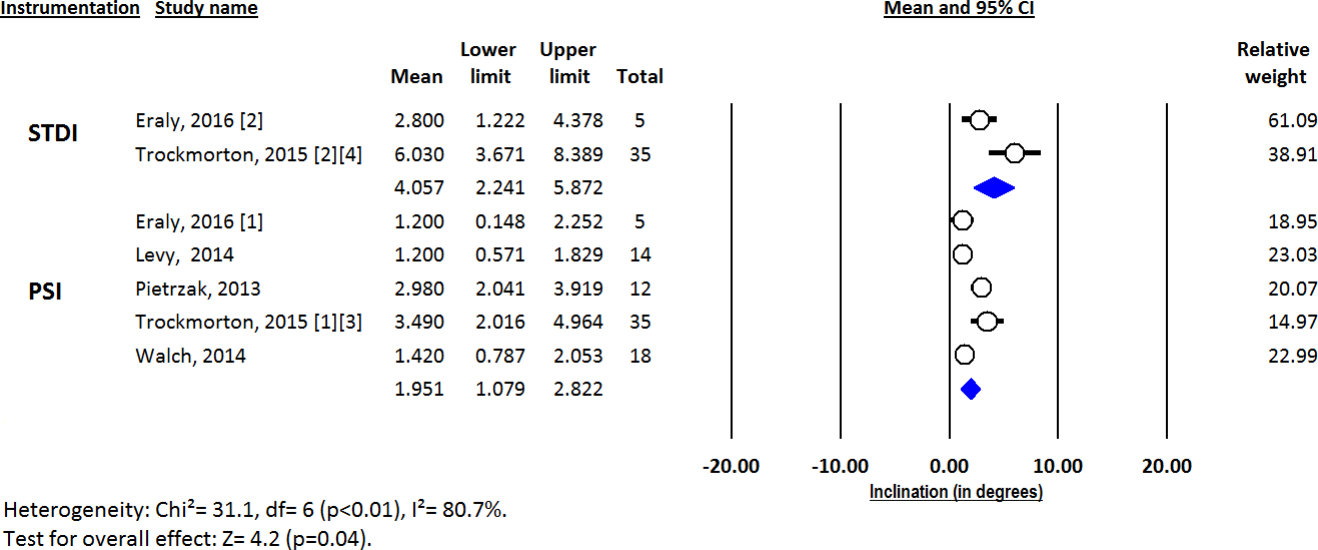
Forest plot of inclination deviation from the pre-operative planning, for cadaveric studies.

**Fig 9:**
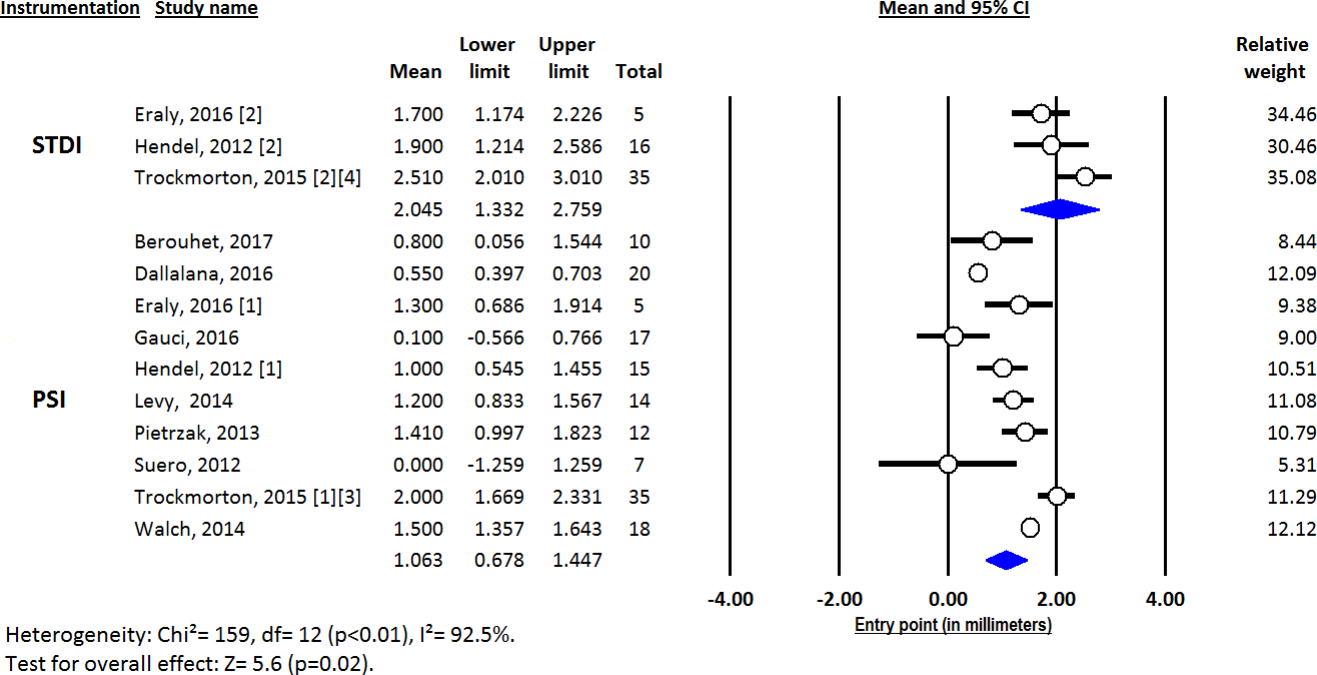
Forest plot of entry point deviation from the pre-operative planning, for all included studies.

**Fig 10:**
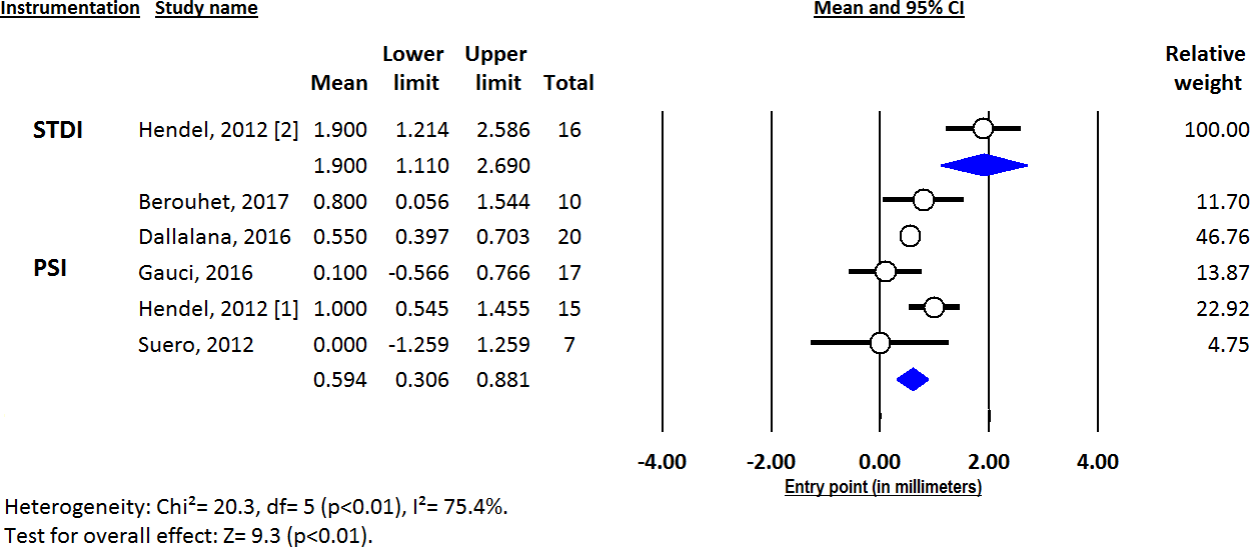
Forest plot of entry point deviation from the pre-operative planning, for clinical studies.

**Fig 11:**
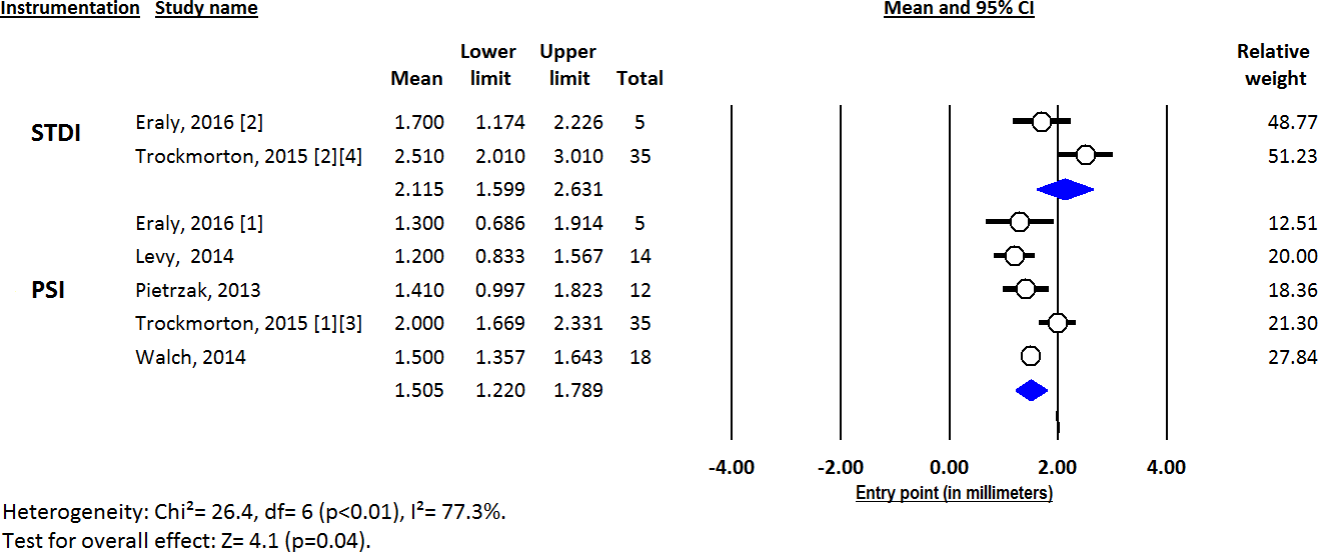
Forest plot of entry point deviation from the pre-operative planning, for cadaveric studies.

**Fig 12:**
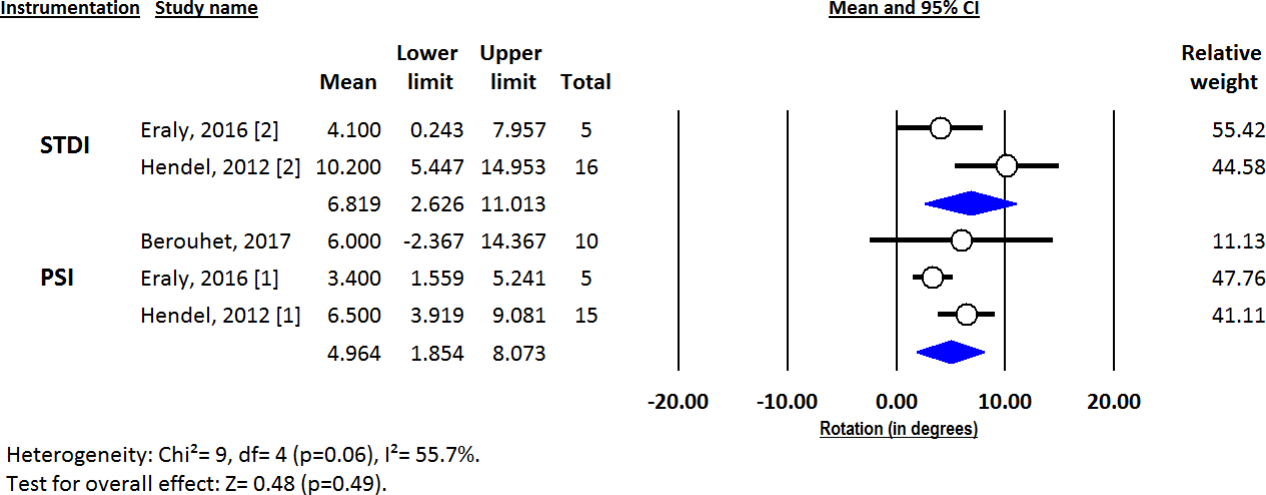
Forest plot of rotation deviation from the pre-operative planning, for all included studies.

Deviations from pre-operative planning for version (Figs 3–5), inclination (Figs 6–8) and entry point (Figs 9–11) were significantly lower with the PSI method than with the STDI method (p<0.01; p<0.01; and p = 0.02 respectively). These differences were systematically found in all analyses (cadaveric, clinical and global). The difference between the two methods was not significant where deviation for rotation was concerned (p = 0.49) (Fig 12).

**Fig 13:**
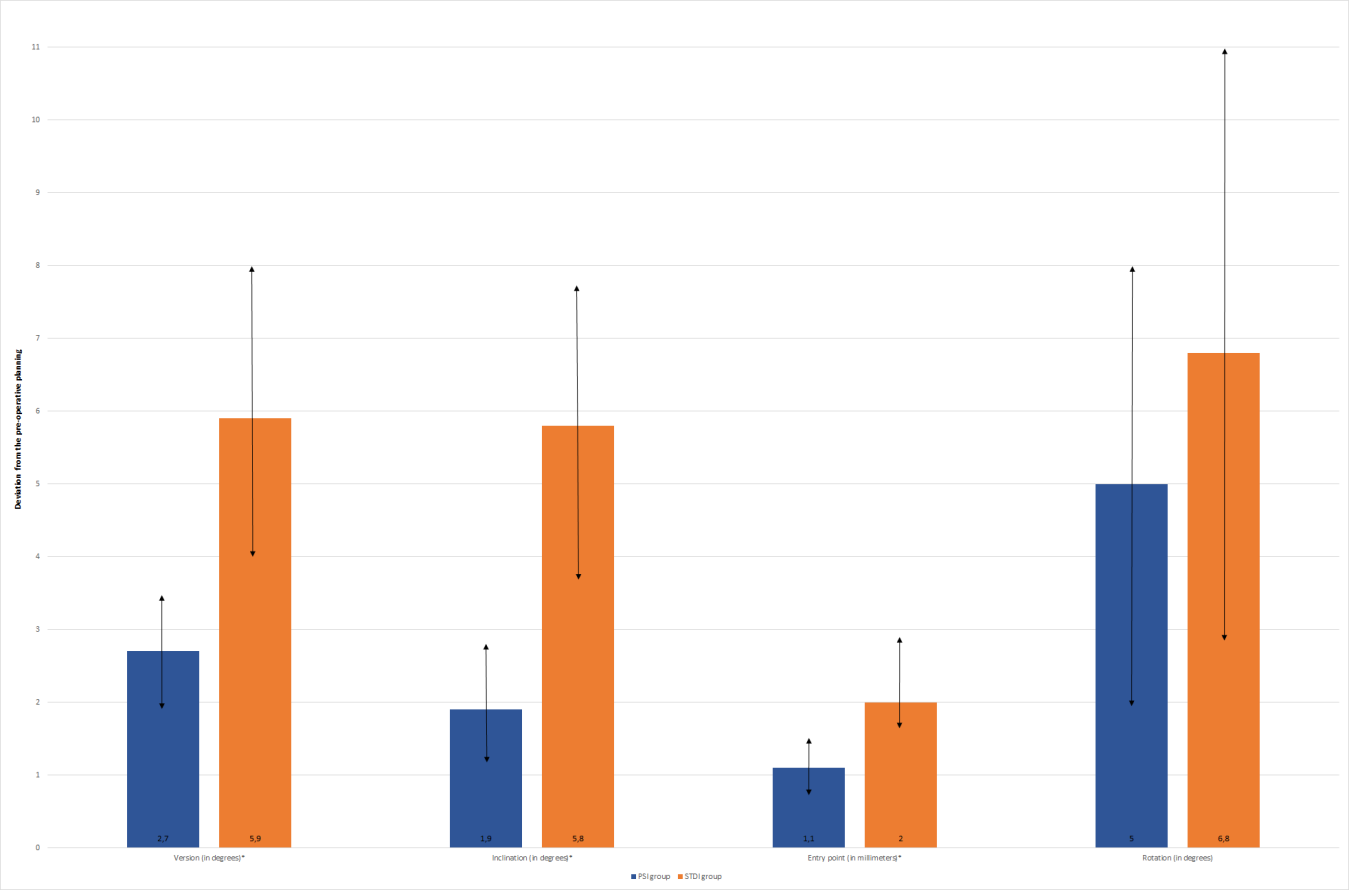
Mean deviations from the pre-operative planning for each glenoid parameter included in the study (* = significant difference; ↕ = range of results). (The mean difference between PSI and SDTI were approximately 3.15°, 3.89°, 0.98mm and 1.86° for version, inclination, entry-point and rotation respectively).

Deviations from the pre-operative planning were as follows (Fig 13):

- for PSI: 2.73° (SD = 0.48) for version; 1.88° (SD = 0.41) for inclination; 1.06mm (SD = 0.20) for entry point; and 4.96° (SD = 1.59) for rotation.
- for STDI: 5.88° (SD = 1.10) for version; 5.78° (SD = 0.98) for inclination; 2.04mm (SD = 0.40) for entry point; and 6.82° (SD = 2.14) for rotation.

68.6% (36/51) of component were classified as outliers when using the SDTI method, compared to 15.3% (18/118) with the PSI method (p = 0.01).

**Table 2:** Summary of the data provided by each study regarding the questions of the review.

Study	Number of cases (PSI group / STDI group)	Level of evidence	Conclusions 1. comparison between PSI and STDI methods to reproduce pre-operative planning. 2. assessment of PSI accuracy and reliability to reproduce pre-operative planning. 3. number of outliers.
Hendel et al 2012	15 / 16	I	1. PSI > STDI for I*, medial-lateral offset*, and for all parameters when native retroversion is up to16°*. 2. good accuracy and reliability for EP-V-I-R. 3. 27% with PSI—75% with STDI.
Suero et al 2012	7 / 0	IV	1. NA. 2. good accuracy and reliability EP-V-I-R. 3. NA.
Pietrzak 2013	12 / 0	Basic science	1. NA. 2. good accuracy and reliability for EP-V-I. 3. 0% with PSI.
Subramanya et al 2014	7 / 0	IV	1. NA. 2. good accuracy and reliability for V-I. 3. 0% with PSI.
Levy et al 2014	14 / 0	Basic science	1.NA. 2. high accuracy and reliability for EP-V-I. 3. NA.
Walch et al 2014	18 / 0	Basic science	1. NA. 2. good accuracy and reliability for EP-V-I. 3. 0% with PSI.
Trockmorton et al 2015	35 / 35	Basic science	1. PSI > STDI for angular deviation* (for all), and I*-V* (for aTSA). 2. good accuracy and reliability EP-V-I. 3. 17% with PSI—66% with STDI.
Eraly et al 2016	5 / 5	Basic science	1. PSI > STDI for angular deviation*. 2. good accuracy and reliability for EP-V-I-R. 3. NA.
Gauci et al 2016	17 / 0	IV	1. NA. 2. good accuracy and reliability for EP-V-I. 3. NA.
Dallalana et al 2016	20 / 0	IV	1. NA. 2. good accuracy and reliability for version, inclination, entry point. 3. 5% with PSI.
Lau et al 2017	11 / 0	IV	1. NA. 2. poor accuracy and reliability for V-I. 3. 64% with PSI.
Berouhet et al 2018	10 / 0	IV	1. NA. 2. good accuracy and reliability for version, inclination, entry point, but not for rotation and reaming depth. 3. NA.

PSI: Patient Specific Instrumentation; STDI: Standard Instrumentation; EP: Entry Point; V: Version; I: Inclination; R: Roll; angular deviation = version + inclination

*: significant difference, NA: Not Available.

## Discussion

This study is the first review with meta-analysis of the effectiveness of PSI on glenoid component positioning during TSA. PSI significantly improves the positioning of the glenoid, especially when looking at outlying components.

This meta-analysis has several limitations. First of all, heterogenous studies were included to increase the strength of the overall analysis. This heterogeneity is a consequence of: the study designs (randomized studies or retrospective case series with aTSA and/or rTSA); and the fact that the procedures were performed on humans and cadaver specimens. This bias was controlled because the surgical technique (delto-pectoral approach, one central guidewire), the outcomes (various angulations of implant), and the measurement method (CT scan) were similar in all studies. Moreover, the outcomes based on deviation from the pre-operative planning limited the risk of heterogeneity of measurements due to the two different kinds of processes (fully automated software versus manual assistance software for segmentation and glenoid measurements calculation). Finally, the methodology of the meta-analysis (weighting of results based on the power of each study, a separate analysis of clinical and cadaveric studies and a combined analysis of all results) showed a good level of consistency in the results. This is supported by the studies in this review with the best design and highest quality level both clinical and cadaveric. Concerning the secondary outcome about direct comparison between STDI and PSI results, only 3 out of 12 studies (1/7 clinical studies and 2/5 cadaveric studies) performed this analysis, and consequently we got limited data on STDI results. Even if these 3 studies involved more than 80% of the included population (183/227 patients or cadavers), this led to a potential bias in favour of PSI, and consequently these results should be considered as exploratory. The use of only English language papers could have incurred a selection bias, although no articles in any other language were found during various database searches. Another limitation is that each individual article included in the systematic review is also subject to its own biases. These inherent biases have the potential to create a downstream effect in the synthesis of the conclusions drawn in this review. Although slightly outside the scope of this study, a further limitation is that no clinical study has been published on the impact of PSI on clinical outcomes and long-term survivorship.

Even if glenoid component positioning is considered to be a major factor in survivorship [10,11], there is still a debate about the ideal position. Historically, literature on the subject only focused on two-dimensional positioning including version (0–15° of retroversion recommended) and inclination (0–10° of inferior inclination recommended), but the use of pre-operative 3D-planning [39] proved that rotation, entry point and reaming depth are also very important parameters to consider. Finally, other authors [40–43] determined the normal pre-morbid glenoid anatomy using a software based on the pre-operative CT scan and constructed the implant positioning parameters accordingly. The issue is that not all authors agree on how to measure glenoid parameters, some preferring fixed anatomic landmarks based on the work of Friedman and Churchill [44,45], whereas others preferring whole scapula body landmarks and mathematical principles [46]. This last point is consistent with the result that two kinds of processes (software and measurement calculations) were used across the different studies.

Whatever the objective, the most important outcome is to accurately reproduce a preoperative plan, assuming the plan itself is accurate and optimised. Surgically this is complex because glenoid exposure is a technically difficult step [47,48]) and there is no reliable intra-operative landmark to determine glenoid morphology and scapular plane [49]. Surgeon’s ability to accurately position a glenoid component with STDI [15,16,28,50,51] is limited, with a mean deviation of approximately 5–10° for version and inclination. These values are affected by surgeon inexperience [36] and variations in bone loss in the patient [52,53].

The results of this review, with 11/12 studies giving a rather favorable general point of view of PSI, show that it is more accurate than STDI for all glenoid parameters (although not significant for rotation, probably due to a lack of power analysis, with only three studies assessing this parameter) (Fig 13). The differences between PSI and STDI could seem very low in value (and maybe not clinically relevant) with respect to the mean for each parameter (3.15° for mean version deviation, for example) but when taking into account the number of extreme values or outliers for global positioning (controlling all parameters together), the impact of PSI on improving accuracy and reproducibility is clear (the SDTI method produced 68.6% component outliers compared to only 15.3% with the PSI method (p = 0.01)). This conclusion was also confirmed by a study from Iannotti [14] on 197 plastic bone scapula models (with arthritic deformities), which found that, overall, PSI reduced the risk of deviating by 5° or more from the pre-operative plan. The risk was reduced by 90% (95% CI, 75% to 96%) for version and 96% (95% CI, 90% to 99%) for inclination (p < 0.001 for both). Finally, Hendel [28] also demonstrated that the impact of PSI compared to STDI was even more important when pre-morbid glenoid deformity was complex (more retroverted, for example).

The difference between the pre-operative planning and the final result when using PSI is probably due to the limitations of the software in creating a perfect glenoid mold (initial segmentation process), and to the surgeon’s ability to both find the same landmarks to correctly seat the guidewire on the glenoid surface and to ream in line with the guide pin (bending or pushing the guide pin with the reamer can cause further unsuitable reaming) [17,54]. This was illustrated in three cadaveric studies [35,37,38], which only measured the guide pin position and not the final glenoid component position, with mean differences of approximately 0.11mm for the entry point and 2.74° and 2.95° for version and inclination respectively.

Future developments will focus on three main areas. Firstly, the development of personalized guides, to include systematically all glenoid component parameters (version, inclination, rotation and reaming depth). Secondly, the development of pre-operative imaging software with better segmentation to lessen the problems of seating the guidewire on the glenoid surface. Thirdly, improvements in teaching the PSI method, with more user-friendly software and better understanding of bone structures (informing the decision whether to preserve calcified and ossified parts of the glenoid rim or labrum before seating the guidewire).

## Conclusion

This review supports the idea that PSI, when compared with STDI, improves glenoid component positioning during TSA within few degrees or millimeters. Nevertheless, further innovation and learning is necessary to decrease the number of poorly positioned implants, which is the main etiology of shoulder prosthesis failure. Clinical studies are also needed to confirm the hypothetical long-term benefits.

## Supporting information

S1 Table PRIMSA checklist. (DOC)

S1 Text Example of electronic search strategy on Pubmed database. (DOC)
