# Supplementary figures and images for: Use of Patient-Specific Instrumentation (PSI) for glenoid component positioning in shoulder arthroplasty. A systematic review and meta-analysis
Source: PLoS One. 2018 Aug 22;13(8):e0201759. doi: 10.1371/journal.pone.0201759 (PMC6104947; doi:10.1371/journal.pone.0201759)

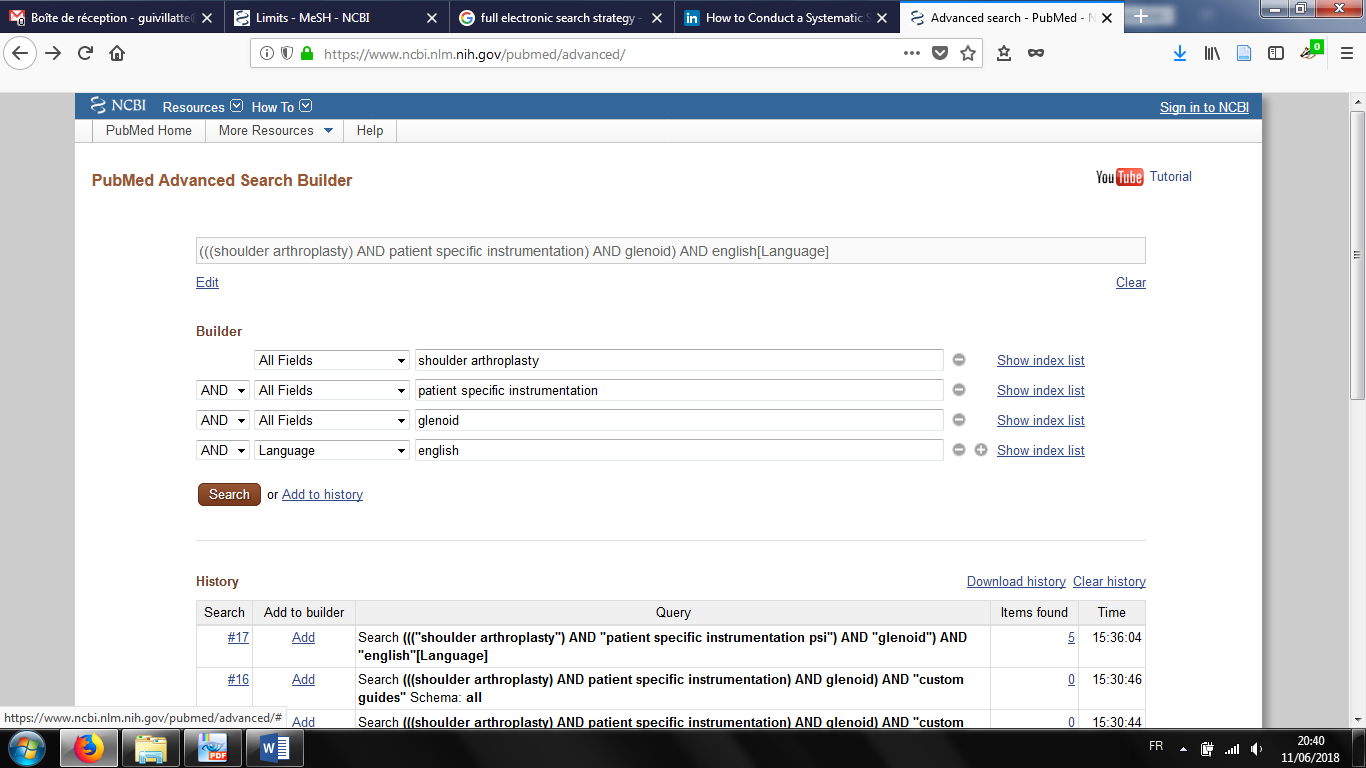

Supplement: S1 Text — (DOC) [file pone.0201759.s002.doc]
